# Should assisted dying be legalised?

**DOI:** 10.1186/1747-5341-9-3

**Published:** 2014-01-15

**Authors:** Thomas D G Frost, Devan Sinha, Barnabas J Gilbert

**Affiliations:** 1 Lincoln College, Turl Street, Oxford OX1 3DR, UK; 2 Brasenose College, Oxford OX1 4AJ, UK; 3 Green Templeton College, Woodstock Road, Oxford OX2 6HG, UK

**Keywords:** Assisted dying, Legalisation, Physician-assisted suicide, Euthanasia

## Abstract

When an individual facing intractable pain is given an estimate of a few months to live, does hastening death become a viable and legitimate alternative for willing patients? Has the time come for physicians to do away with the traditional notion of healthcare as maintaining or improving physical and mental health, and instead accept their own limitations by facilitating death when requested? The Universities of Oxford and Cambridge held the 2013 Varsity Medical Debate on the motion “This House Would Legalise Assisted Dying”. This article summarises the key arguments developed over the course of the debate. We will explore how assisted dying can affect both the patient and doctor; the nature of consent and limits of autonomy; the effects on society; the viability of a proposed model; and, perhaps most importantly, the potential need for the practice within our current medico-legal framework.

## Introduction

Over the past two centuries, the United Kingdom has experienced rapid population growth associated with a substantial decline in mortality from acute infectious diseases and poor nutrition [1]. As the average life expectancy has increased, so too have the rates of debilitating chronic illness – particularly coronary artery disease and cancers [2]. These diseases require years of treatment instead of the mere days to weeks that medicine once operated within [2]. Although healthcare systems have sought to adapt to such changes, aiming to prevent and treat such disease wherever possible, debate has arisen regarding those patients in the latter stages of chronic, incurable, terminal conditions [3,4]. Moreover, there is increasing recognition that the patient must be at the centre of health care decision-making, such that outcomes must be tailored to their individual needs and views. By extension, assisted dying might seem a logical step to help achieve these goals within the realm of end-of-life decision making [5]. Several jurisdictions, notably Oregon (1997) and the Netherlands (2001) have already legalised assisted dying in some form. These factors have contributed to ongoing legislative discussions within Parliaments for almost a decade, with current opinion polling suggesting a majority of medical practitioners and the public in favour of physician-assisted suicide [6].

### Viability of assisted dying in practice

In the UK, a model for assisted dying has been developed from the legal structure found within the Assisted Dying Bill introduced by Lord Falconer in the House of Lords in 2013 [7]. Assisted dying could only be considered under circumstances in which a patient of legal age is diagnosed with a progressive disease that is irreversible by treatment and is “reasonably expected to die within six months” [7]. Registered medical practitioners would make such decisions for patients with terminal illnesses. Addressing the technicalities of ‘assisted dying’ requires distinction between ‘physician-assisted suicide’ (offering patients medical actions or cessation of actions by which they can end their own life) and ‘euthanasia’ (whereby the medical practitioner actively induces death). In light of the strong hostility of the medical profession towards active euthanasia, this proposed model, as with previous attempts to legalise assisted dying, permitted only the former [8-10].

However, there is concern that such distinction may be unrealistic in practice because medical practitioners could find themselves with a patient who had failed to successfully end their own life and was subsequently left in a state of greater suffering. Were such a patient no longer able to give consent, a heavy burden would then be placed on the physician regarding how to proceed. Moreover, the practice of physician-assisted suicide might be deemed discriminatory, for example by giving only patients with good mobility control over their own method of death.

The Assisted Dying Bill 2013 included the provision that any terminal prognosis must be confirmed and attested by a second registered practitioner. The strictness of such criteria has parallels to a similar double-physician requirement when procuring a legal abortion under the 1967 Abortion Act. The stated aims of the provision in both cases are as follows: first, to check the accuracy of the prognosis upon which the decision was being made; second, to ensure that the situation meets the required criteria; and third, to check that such a decision was taken by the patient after full consideration of all available options [11,12]. By having a second independent doctor, the legislation ensures that all three checks are met without prejudice or mistake.

Problematic for any protocol for assisted dying is the fact that estimates of life expectancy in terminal prognoses are erroneous in 80.3% of cases [13]. Furthermore, the accuracy of such prognoses deteriorates with increased length of clinical predicted survival. Forecasts of survival times are based largely on past clinical experience, and the inherent variability between patients makes this more of an art than a science. This brings to concern both the accuracy of any prognosis meeting the six-month threshold and the validity of requests for assisted dying based partly or wholly on predicted survival times. Whilst the majority of errors in life expectancy forecasts are a matter of over-optimism and hence would not affect either of those two concerns, many cases remain unaccounted for. Overly pessimistic forecasts occur in 17.3% of prognoses; hence we must decide whether the one in six patients making a decision based on an inaccurate prognosis is too high a cost to justify the use of this system. Patients requesting an assisted death often cite future expectations of dependency, loss of dignity, or pain [14]. If the hypothetical point at which the progression of their illness means they would consider life to be not worth living is not, as informed, mere weeks away but in fact many more months, then this information would have resulted in a different decision outcome and potentiated unnecessary loss of life.

Whilst the presence of a second doctor would be expected to mitigate such forecasting errors, the anchoring bias of the initial prediction may be enough to similarly reduce the accuracy of the second estimate. It is prudent to question the true independence of a second medical practitioner, and whether this second consultation could become more of a formality, as has now become the case with abortion [15].

Another challenge for an assisted dying system would be to recognise whether patients requesting death were legally competent to make that decision. Consider that any request for suicide from a patient with clinical depression is generally categorised as a manifestation of that mental disorder, thereby lacking capacity. It is arguably impossible to separate out the natural reactions to terminal illness and clinical depression. Indeed, there is evidence that major depressive disorders afflict between 25% and 77% of patients with terminal illness [16,17]. Any protocol for assisted dying must first determine what qualifies as a ‘fit mental state’ for a terminal patient.

### The need for assisted dying

It could be argued that a doctor’s fundamental duty is to alleviate forms of suffering in the best interests of the patient. The avoidance of physical pain, as an obvious manifestation of suffering, might explain why assisted dying would be both necessary and within the duties of a doctor to provide. The evolving principle in common law known as the ‘Doctrine of Double Effect’ offers a solution to this problem [18]. This legal judgement stated that “[a doctor] is entitled to do all that is proper and necessary to relieve pain even if the measures he takes may incidentally shorten life”. This entails that a protocol already exists for patients searching for an escape from chronic pain. Furthermore, numerous retrospective studies have revealed very little correlation between opioid dose and mean survival times: one study of over 700 opioid-treated patients found that the variation in survival time from high-dose opioid treatment is less than 10% [19-21]. It can therefore be said that pain alone, if appropriately managed, should never be cause for considering assisted dying as an alternative.

By contrast, the ‘Doctrine of Double Effect’ might be seen as a subjective interpretation that has been applied unequally due to a lack of specialist training or knowledge [22]. Despite this, the principle can be easily understood and poor awareness can be remedied by improvements in medical education and standardisation of protocols. Moreover, should we choose to accept arguments for assisted dying that are based upon inadequate administration of pain medication, we set a precedent for conceding shortcomings in palliative care and other end-of-life treatments. Offering hastened death could become an alternative to actively seeking to improve such failings.

Whilst much has been made of the ‘pain argument’ here, the call for assisted dying is rarely this simple. Many patients also suffer a loss of dignity, often due to their lack of mobility – the inability to relieve oneself without help is a potent example. Beyond this are additional fears of further debilitation and the emotional costs of dealing with chronic illness, both for the patient and for their relatives and friends. A study of terminal patients in Oregon showed that these were the most significant reasons behind requests for assisted suicide, the next commonest reason being the perception of themselves as a ‘burden’ [14]. Clearly, we could seek to provide balanced, compassionate medical care for these patients, and still fail to address these points.

Developments in healthcare and technology may reduce this emotional burden, but remain an imperfect solution.

### Rights of patients and limitations of their autonomy

J. S. Mill’s pithy dictum describes autonomy as follows: “over himself, over his own body and mind, the individual is sovereign” [23]. Not only has the sanctity of bodily autonomy profoundly influenced the development of liberal democracies, it has also provoked a holistic shift in making our healthcare systems more patient-centred – “care that meets and responds to patients’ wants, needs and preferences and where patients are autonomous and able to decide for themselves” [5]. The ethical principle of controlling the fate of one’s own body is inherently relevant to the debate on assisted dying. It is difficult to reconcile that citizens may have the right to do almost anything to and with their own bodies– from participating in extreme sports to having elective plastic surgery – yet a terminal patient cannot choose to avoid experiencing additional months of discomfort or loss of dignity in their final months of life.

Expectation of individual liberty has been codified in law. The right to bodily autonomy has been interpreted to be included under Article 8 - the right to privacy - of the European Convention on Human Rights (ECHR) and subsequently the Human Rights Act (HRA) [24,25]. Moreover, the ECHR underpins the right of individuals to ‘inherent dignity’ [26]. Hence, if an individual feels that dignity is unattainable due to the progression of a terminal illness, then taking recourse though assisted dying ought to be a legitimate option.

Conversely, there are two notable oversights in this interpretation of a right to assisted dying as an extension of the principles of bodily autonomy:

First, it would be wrong to view individual liberty as absolute. The HRA allows for exceptions to Article 8 on grounds of ‘health or morals’ [25]. The principle of autonomy is not inviolable. Governments have limited such privileges for the protection of individuals and society, for example by criminalizing the use of recreational drugs or the selling of one’s own organs. The preservation of life by denying assisted dying could fall within this category.

Second, the right of autonomy is not necessarily intrinsic to human beings but, as Kant argued, is dependent on our ‘rational nature’ [27]. This concept sees autonomy as an exercise of ‘evaluative choice’ [27], requiring rationality on the part of individuals to appreciate the nature of options and their consequences. To achieve true autonomy, there must be sufficient information to make those rational decisions; this is the basis of informed consent and why it is a fundamental duty of a doctor to offer a patient an informed series of treatment options [28]. The logistical issue is that doctors are unable to advise patients regarding the point at which their situation becomes less preferable to being dead. No doctor (or individual) has any knowledge or experience of what ‘death’ may be like. Hence, in this case, the idea of exercising true autonomy through informed consent might be considered meaningless.

Legalising assisted dying by attempting to establish an absolute right to bodily autonomy may undermine other individual and group rights. Vulnerable patients may feel pressured into assisted dying because of social, emotional, or financial strains placed on family and/or friends. This is exemplified by the trend showing that the proportion of patients stating ‘relief of burden’ on others as the reason for requesting assisted dying has risen from 17% to 25% in Oregon since legalisation [29]. One could even consider the risk of assisted dying becoming an expected choice rather than a free one. Thus, assisted dying may erode the elemental right to life of terminal patients as the value of their life becomes tied to relative costs to society and to those around them.

Moreover, by creating one class of individuals for whom life is expendable, that particular view may be extended by society to all groups possessing such attributes (e.g. the permanently disabled). There would be a definite risk to the rights of these vulnerable groups in the form of society being less willing to provide for their health and social care.

It is often raised that the limited legalisation of assisted dying would inevitably become extended in scope, but this is not necessarily a flaw. Even if the right to determine the manner of death were later extended to a wider group of people, posterity may reflect positively on such a change, just as extending the franchise to women ultimately led to legislation demanding equal pay.

### Effect on health professionals and their role

‘To act in the best interest of the patient’ is often cited as a central duty of the doctor [28]. This concept of ‘best interest’ guiding the doctor’s action has seen the development of two important ethical principles: beneficence and non-maleficence. Beneficence mandates that the actions of the doctor must be aimed to bring about benefit (clinical improvement) for the patient, usually measured in terms of reduced morbidity or mortality; non-maleficence requires that the doctor not carry out treatment that is likely to cause overall harm the patient [30]. These traditional ethical imperatives on a doctor both conflict with intentionally hastening the death of a patient, and a resolution of this tension would require redefining what constitutes ‘acting in the best interest’.

A further dimension is the potential reluctance of health professionals to engage in a practice that contravenes their own ethical beliefs, particularly as this would affect doctors who never entered training in the knowledge that assisting patients to die would be an expected duty. This is certainly no argument against the introduction of assisted dying; indeed, a recent survey of a cohort of NHS doctors found that 46% would seriously consider requests from patients to undertake steps to hasten death [31]. It merely expresses the point that any early model would have to account for the fact that an initial 54% of the doctors in the NHS would be required to advise qualifying patients of assisted dying as a legitimate option, despite disagreeing with it in principle.

Furthermore, doctors who agree ethically with this practice may find themselves facing conflicts of interest. It is expensive to treat chronically ill patients, particularly in the final months of life [32]. Moreover, it would be difficult for commissioners to ignore the fact that the sustained treatment of one individual could deprive many others from access to surgery or access to novel drugs. Such an argument does *not* suggest that doctors or any other hospital staff would treat this practice without appropriate respect or care; rather it acknowledges the need for appropriate rationing of care and questions the intentions of service providers. The perception of an ulterior motive could negatively impact patient trust. One survey showed that a reasonable minority of patients (27%) – and particularly particularly the elderly – believe that legalising assisted dying would lessen their trust in their personal physician [33]. The costs of weakened trust in the doctor-patient relationship could far outweigh the benefits of assisted dying, particularly given the importance of trust when treating a chronic patient for an extended period of time.

## Conclusion

There is no doubt that assisted dying would empower some patients to maximise control over the timing and manner of their own death. Such expression of autonomy would surely solidify moves towards a patient-centred approach to healthcare. However, the capacity for such consensual requests remains in doubt. Clinically, the patient’s state of mind and the reliability of diagnostic predictions are of issue; philosophically, the idea of informed consent for death is contradictory. The implications for patients, physicians and society have been weighed extensively within this article. The central tenet throughout has been the balancing of an individual’s right to escape a circumstance that they find intolerable, alongside the consequential changes to their other rights, and the rights and responsibilities of third parties. Ultimately, the challenge is for us as a society to decide where this balance lies.

### About the debate

The Varsity Medical Debate was started in 2008 with the aim of allowing students, professors and members of the polis, to engage in discussion about ethics and policy within healthcare. Utilising the age-old rivalry between the two Universities, the debate encourages medical students from both Oxford and Cambridge to consider and articulate the arguments behind topics that will feature heavily in their future careers.

The debate was judged on the logic, coherence, and evidence in arguments, as well as flair in presentation. Although the debaters may not have necessarily agreed with their allocated side, the debate format required them to acknowledge a particular school of thought and present the key arguments behind it. Oxford, who opposed the motion, was awarded the victory in the debate; however, this does not mean that the judges believe that position ought to become public policy.

## Competing interests

The authors declare that they have no competing interests.

## Authors’ contributions

All authors planned and elucidated the layout. TDGF and DS drafted the manuscript which was critically edited and added to by BJG. All authors have read and approved the final draft.

## References

[B1] Colgrove J The McKeown thesis: a historical controversy and its enduring influence Am J Public Health 2002 92 725 729 10.2105/AJPH.92.5.725 11988435 PMC1447153

[B2] Yach D Hawkes C Linn Gould C Hofman K The global burden of chronic diseases: overcoming impediments to prevention and control J Am Med Assoc 2004 291 21 2616 2622 doi:10.1001/jama.291.21.2616 10.1001/jama.291.21.2616 15173153

[B3] Lankhorst EK Spreeuwenberg C Nolte E, Cécile K, Martin MK Managing Chronic Conditions. Experience in Eight Countries European Observatory on Health Systems and Policies 2008 The Netherlands: WHO Regional Office Europe

[B4] Hudson PL Kristjanson LJ Ashby M Desire for hastened death in patients with advanced disease and the evidence base of clinical guidelines: a systematic review Palliat Med 2006 20 693 701 10.1177/0269216306071799 17060268

[B5] Hogg C Patient-Centred Care - Tomorrow’s Doctors 2004 GMC

[B6] Harris D Richard B Khanna P Assisted dying: the ongoing debate Postgrad Med J 2006 82 970 479 482 doi:10.1136/pgmj.2006.047530 10.1136/pgmj.2006.047530 16891435 PMC2585714

[B7] House of Lords assisted Dying Bill House of Lords 2013 http://www.publications.parliament.uk/pa/bills/lbill/2013-2014/0024/2014024.pdf (accessed 23 October 2013)

[B8] House of Lords Assisted Dying for the Terminally Ill Bill House of Lords 2005 http://www.publications.parliament.uk/pa/ld200506/ldbills/036/2006036.pdf (accessed 15 Feb 2006)

[B9] Sanders K Chaloner C Voluntary euthanasia: ethical concepts and definitions Nurs Stand 2007 21 35 41 44 10.7748/ns2007.05.21.35.41.c4554 17515151

[B10] Lee W Price A Rayner L Hotopf M Survey of doctors’ opinions of the legalisation of physician assisted suicide BMC Med Ethics 2009 10 2 10.1186/1472-6939-10-2 19261197 PMC2673229

[B11] House of Commons Science and Technology Committee, Scientific Developments Relating to the Abortion Act 1967 2007 Volume 1 London: tso

[B12] Royal College of Obstetricians and Gynecologists, Campaigns and Opinionshttp://www.rcog.org.uk/what-we-do/campaigning-and-opinions/briefings-and-qas-/human-fertilisation-and-embryology-bill/brie-1

[B13] Christakis NA Lamont EB Extent and determinants of error in physicians’ prognoses in terminally ill patients BMJ 2000 320 469 473 10.1136/bmj.320.7233.469 10678857 PMC27288

[B14] Ganzini L Goy E Dobscha S Oregonians’ reasons for requesting physician aid in dying J Am Med Assoc Int Med 2009 169 5 489 492 doi:10.1001/archinternmed.2008.579 10.1001/archinternmed.2008.579 19273779

[B15] Care Quality Commision. Findings of Termination of Pregnancy Inspections Published July 2012 http://www.cqc.org.uk/media/findings-termination-pregnancy-inspections-published

[B16] Watson M Lucas C Hoy A Oxford Handbook of Palliative Care 2005 Oxford: Oxford University Medical Press

[B17] Fine R Depression, anxiety, and delirium in the terminally ill patient Proc (Bayl Univ Med Cent) 2001 14 2 130 133 16369601 10.1080/08998280.2001.11927747 PMC1291326

[B18] R v Adams [1957] Crim LR 773

[B19] Morita T Tsunoda J Inoue S Chihara S Effects of high dose opioids and sedatives on survival in terminally ill cancer patients J Pain Symptom Manage 2001 21 4 282 289 doi:10.1016/S0885-3924(01)00258-5 10.1016/S0885-3924(01)00258-5 11312042

[B20] Bengoechea I Gutiérrez S Vrotsou K Onaindia M Lopez J Opioid use at the end of life and survival in a hospital at home unit J Palliat Med 2010 100828074323069 doi:10.1089/jpm.2010.0031 10.1089/jpm.2010.0031 20799903

[B21] Thorns A Sykes N Opioid use in last week of life and implications for end-of-life decision-making Lancet 2000 356 9227 398 399 doi:10.1016/S0140-6736(00)02534-4 10.1016/S0140-6736(00)02534-4 10972375

[B22] Preston T Patterson J The rule of double effect N Engl J Med 1998 338 1389 1391 9575058

[B23] Mill JS “On Liberty” in On Liberty and Other Essays 14 15

[B24] Human Rights Act 1998 HMSO

[B25] Judgment on the Merits Delivered by a Chamber. Y.F. v. TURKEY, no. 24209/94, ECHR 2003-IV

[B26] McCrudden C Human dignity and judicial interpretation of human rights Eur J Int Law 2008 19 4 655 724 doi:10.1093/ejil/chn043 10.1093/ejil/chn043

[B27] Savulescu J Autonomy, the Good Life and Controversial Choices 2006

[B28] General Medical Council Good Medical Practice 2013 GMC

[B29] Oregon Department of Human Services. Fifth Annual Report on Oregon’s Death with Dignity act http://egov.oregon.gov/DHS/ph/pas/docs/year5.pdf

[B30] Campbell Medical Ethics 1997 Oxford: Oxford University Press

[B31] Ward B Tate P Attitudes among NHS doctors to requests for euthanasia BMJ 1994 308 1332 10.1136/bmj.308.6940.1332 8019219 PMC2540258

[B32] National Audit Office. End of Life Care Report by the Comptroller and Auditor General. HC 1043 Session 2007-2008 2008 NAO

[B33] Hall M Trachtenberg M Duggan E The impact on patient trust of legalising physician aid in dying J Med Ethics 2005 31 12 693 697 doi:10.1136/jme.2004.011452 10.1136/jme.2004.011452 16319229 PMC1734062

